# Homœopathy, Its Tenets and Tendencies—Theoretical, Theological and Therapeutical

**Published:** 1854-07

**Authors:** 


### REVIEW DEPARTMENT.

### ARTICLE XII.

*Homœopathy, its Tenets and Tendencies—Theoretical, Theological and Therapeutical.* By James Y. Simpson, M. D., F. R. S. E., Professor of Midwifery in the University of Edinburg, &c. &c. First American from the third Edinburg edition. Philadelphia. Lindsay & Blakiston, 1854.

*Homœopathy fairly Represented; a Reply to Professor Simpson's "Homœopathy" Misrepresented.* By Willim Henderson, M. D., Professor of General Pathology in the University of Edinburgh. First American from the last Edinburgh edition. Philadelphia. Lindsay & Blakiston.

About homœopathy, as a medical system, there can be no serious controversy. It has no more necessary connection with the healing art than spiritual rappings with rational theology. The basis of medicine is common sense; the superstructure is observation and experience. Homœopathy has no fixed basis, unless it be want of common sense. Its superstructure is the negative of observation and the contradiction of experience. It is extremely difficult to express in positive language the negative ideas involved in the philosophy of homœopathy. Coleridge would have resorted to the mathematical signs, and by a free use of minuses and cyphers endeavored to convey some picture, if not notion, of the results of an indeterminate analysis of nothing. We leave this process to such of our readers as have a taste for transcendental calculations. We are too practically inclined for the sublimities or profundities of impossible metaphysics. If even a modicum of truth were extortable out of this interminable platitude and fog, we would courageously essay to discover it. But "fleas are not lobsters," and no boiling can redden the nimble parasite into the hues of the simmering crustacean.

Peter Pindar has recorded in heroic verse, the vexations of the great naturalist who lost his temper over the pot of inconvertible insects. We have no wish to exhaust ours in an attempt to get bones out of syllabub or something out of homœopathy.

Nevertheless, homœopathy, if impracticable as a theory, is not worthless as a thing. Though a discussion of its philosophy is but an excursion into the realms of boundless absurdity, its practical recommendations and the conduct displayed under them, are subjects for curious contemplation, and will reward inquiry with amusement, if not with instruction. Of intellectual entertainment, indeed it furnishes great variety, from the mere pleasure of thought to the complex psycho-physical enjoyment of positive fun.

The thing, upon the whole, is wonderfully odd. An elderly lady of our acquaintance, somewhat prone to exuberant adornation of a rather hypermatured person, once appeared in public surmounted by an extraordinary erection of gauze and ribbon, called a turban. A gentleman, very remarkable for imitative ingenuity and constructive skill, after contemplating the magnificent head dress with great interest, observed, "I don't think I could make such a thing. I don't think any body could; but if one should take the materials to a third story window and throw them out, they *might come so.*"

Somewhat of the same kind are our thoughts of homœopathy. Looking at the thing, we don't see how any body could make it. We are sure that no body could do it again. Yet we can conceive that the loose, wild, reckless conceits of a lienteric mind, thrown off without judgment or design, might "come so." That the whole heterogeneous mass of materials, facts and fancies—gauze and ribbon—might take the form of a huge and flashy conglomeration, in which light weight of truth, spun out to cob-web tenuity, might be alligated with fancies and spangled with glittering fallacies, the whole bearing the name of homœopathy, or any other designation which generatoric humbug might elect to bestow upon it.

The most unaccountable thing is, that men should fall down and worship such a creation as a perfect gift of God. The most unaccountable, but by no means the strangest thing, for men have always manifested a tendency to worship monstrosities; a curious propensity to wonder after stupid contrivances. To many men there seems no utterance so fascinating as the braying of an ass, provided it be delivered with sufficient positiveness and energy.

He who sets a trap for men must not be too nice about it. Any refinement of artifice may beget suspicion: at least it is labor thrown away. There is no necessity for ingenuity or skill. There needs nothing but novelty in the proposition, and impudence in the proposer. The former may be more or less absurd—the more so the better; but of the latter, there can be no degrees. The impudence must be perfect. There must be no sensibility to shame: no consciousness of the feeling of a lie, whether the assurance be based upon erroneous conviction of truth or upon a deliberate resolution to be false, it does not matter; but one thing is necessary—that one thing is impudence. With a face hard enough to look shame out of countenance, a man is armed with a power to which numbers will succumb without resistance.

He who would learn what can be done by mere hardihood of assertion, must study the doings of Joseph Smith and Hahneman; of knee-knockers and table-spinners, and ghost-editors, writing bad philosophy in worse English; burying common sense and common decency under the dishonored fragments of mutilated speech.

The Samaritans were not the only people obnoxious to the charge of worshiping they knew not what. The eminence of the homœopaths, however, in perverse credulity, is, that while formerly the extreme of folly led men, like the Athenians, to raise a temple to a god of whom they knew nothing, the homœopaths raise theirs to one whom they know to be nothing. They do not merely act without reason, but directly against it. They undertake to make a negative, intensely positive; to bring nothing into the field against every thing; to compound it, swallow it, trace the consequences and register the deeds of it. Nay, they profess to dilute it, nothing, into inconceivable nothingness, and then, out of the nonentity of annihilation, infinitesimally annihilated, to educe potencies before which powers are powerless. *Splendide mendax!* Magnificent humbug!

Should a man advertise to extract sunbeams from cucumbers, he would be a fool. He would deal in the palpably absurd: in the little go "of humbug." A genius carries absurdity into the impalpable regions; pushes humbug to the centesimal dilutions; brings down a fog out of the immensities, and picks pockets in the darkness he has filibustered from chaos.

Will men ever tire of playing at the game of fox and geese?

The two books under review are component parts of a quarrel and controversy between two of the medical professors of the University of Edinburgh, Drs. Simpson and Henderson, the latter of whom, for reasons best known to himself, has become a champion of the delectable system of mystic medicine, called homœopathy, and yet, most disgracefully to the governors of the school, clings to his professorship. Dr. Simpson's book cannot be considered a systematic investigation of homœopathy. Such a thing is an impossibility. One might as well investigate a fog from the German ocean. Dr. Simpson only publishes some reflections upon various theoretical and practical points in the "system," but these reflections are pertinent and shrewd. They expose the charlatanry of Hahneman and his disciples with boldness and skill, and though the author cannot hope to cure a homœopathist of his folly by any force of logic, seeing that to be a homœopathist is to ignore logic as a legitimate route to truth, nor to shame him out of it by exposing the absurdity of the doctrine and practice, since to the homœopath absurdity is a recommendation, yet the book will do good by affording to the uninfected, yet unguarded, of the community, some true notion of the physical and theological principles of the *soi disant* medical philosophers. It will also afford amusement to the *Democriti* who can laugh at human folly, for surely it never presented itself under funnier aspect, than in the exhibition of a grave, learned, wig'd professor of medicine, putting into the mouth of a grown up man, for the cure of his disorders, a sugar plum of the size of a mustard seed, *medicated* by a drop of a solution of metallic oxyd, taken recently from the Red sea, in which one of Pharoah's shoe buckles was introduced some thousands of years ago, or by some equivalent potency.

We have not time to notice in detail the several subjects introduced by Prof. Simpson. To do so, indeed, would make it necessary to write a book as large as his own, for his statements are made with great conciseness.

Of the theological presumptions of Hahneman and his disciples, Dr. Simpson gives some curious, and to many, novel information.

Men who feel unable to sustain their pretensions to dictate to others, by convincing their understanding, are very prone to appeal to their faith. Hahneman spoke of his discovery, as he called it, as an interference of the "Creator and Preserver of mankind." His disciples speak of him as a "messenger from heaven," "the new evangelist," as "the most inspired of discoverers." We are told that through him "christian science became universal, and redemption descended from the dominion of sentiment to that of the ideas and of intelligence." "Homœopathy is not a science merely, but also, for those who comprehend it, a sublime devotion, a form of religion, a rainbow of divine union, holding out to mankind the promise of speedy regeneration."

We do not profess to be able to interpret the above theological dogmas into comprehensible English. Of thus much, however, we are assured, that if the blessings of this new revelation are restricted to those who "*comprehend homœopathy*," they will be far from "universal" in their diffusion. As to the practical application of the spiritual regenerative force embodied in homœopathy, we should certainly have been at utter loss but for an English expositor, a reverend gentleman, who being more familiar we may hope with the spiritual than the carnal department of the heavenly science, has zealously opened to us the new revelation of the modus operandi of redemption. This gentlemen is Rev. Mr. Everest, rector of Wickwar, who, in a sermon preached in aid of a Hahnemanic hospital, takes occasion to open the new gospel to the world.

Mr. Everest thinks, that considering the physical and moral blessings comprised in homœopathy, it is hardly to be supposed that the Holy Scriptures can be silent about it, and upon examination he sees, sure enough, as any body may see, that a great fundamental dogma of Hahneman is clearly set forth there, and given an application vastly more extensive and sublime.

This great discovery of Hahneman was that chronic disorders, in which are included proclivity to disease as well as diseases themselves, are all caused by *psora*, or the principle of cutaneous eruptions; the itch infection, he seems to have had particularly in view. The list of maladies he ascribes to this cause is long and horrible, including mania, melancholia, epilepsy, convulsions, softening of bones, cancer, jaundice, gout and hæmorrhoids, deafness and blindness, bleedings and barrenness, &c., &c., too tedious to mention. So much for the carnal consequences of Adam's itch. Itch *eruptive.*

Now for the spiritual consequences. Itch *corruptive.*

Mr. Everest finds the clue to spiritual psora in the moral meaning of ancient leprosy, the type of sin, and in the miraculous cures thereof by our Lord and his disciples. He assumes that the principle of psora is, in its workings upon the human frame, productive of mental and moral effects so complicated with the proper spiritual corruption that God only can distinguish them. Of course to man then they are confounded, and present themselves as identical, and as such they evidently presented themselves to the spiritual discernment of Rev. Mr. Everest. He continues the list of psoraic consequences as prepared by Hahneman, by adding "dark passions, furious lusts, stubborn obstinacies, scowling tempers, gloomy revenges, jealousies, fretfulness, ill humor, and reluctance to bear patiently the burdens which the Lord lays on man." "The tendency to disorder of the functions aggravates the tendency to sin. The chronic taint in the constitution increases the chronic proneness to sin, which Adam left us. The physical leprosy of the flesh unites with the moral leprosy of the soul. It is this combination of the two, aided often by stimuli, and almost always by large doses of violent inappropriate medicines antecedently given, which festers in your jails, rots in your hulks, seethes in your lanes and alleys, and bubbles up in crime, madness and eccentricity all over your land. This it is which makes your atheist on the one hand, your bigot on the other." "Irreligion is the daughter of internal disorder or disease," and hence he argues that the old system of medicine '*was of no use or value as an aid to conversion.*' But under homœopathic treatment, the itch being expelled, Mr. Everest predicts that "the holy and saving truths of the gospel will be admitted into the heart and *never fail then* to influence the life."

These doctrines are not the peculiar deductions of Rev. Mr. Everest. They are approved and endorsed by "various noblemen, gentlemen and homœopathic physicians," and the sermon was declared with much other commendation, by the editor of the Homœopathic Times, to be "a great achievement." And so it was. Who before Mr. Everest ever had any clear conception of original sin. Who before, among all the acute theologians who wore out their powers upon this perplexing subject, ever caught a glimpse of the sublime and simple truth, that Satan is a great acarus, which having laid in the souls and skins of men the prolific eggs of itch and sin, is now undergoing appropriate destruction in a lake of brimstone! Who again of preachers, ancient or modern, before Mr. Everest, perceived the true obstacle to man's conversion? Did Moses know that Pharoah's heart was hardened by the "itch of Egypt," making him "reluctant to bear patiently the burden which the Lord laid on him?" Did Jeremiah ever dream that the "obstinacy" of the Jews would have given way to sulphur ointment, or that in default of submission he might medicate them to a state of moral docility by a grain of the antidote thrown into the aqueduct of Jerusalem? Who before Mr. Everest had any correct notion of training up a child in the way he should go? How much anxiety, and patience, and lining and precepting will be saved by physicing sin out of the hearts of the rising generation? This, however, without probably any clear idea of the refined and beautiful philosophy of the thing, was practically enforced by Mrs. Squeers, of Dotheboy's Hall, who gave the boys brimstone and treacle before breakfast to purify their blood and prevent carnal appetite! What an affecting exhibition it would be to see the Rev. Mr. Everest at work purifying his congregation. The Sunday services opened by a short exposition of the moral and corporeal invasion of psora. The symptoms of its presence enumerated. Antidote pellets served by the deacons. The Sunday school children, each supplied from the "school spoon" with the preparation of piety—the Rev. Mr. Everest himself, swallowing a potency, as a preservative from the evil influence, and then, all being effectually brimstoned; all obstinacy and ill humor, and folly and impatience of control, being removed, pouring the undiluted truth of Christianity into willing hearts. And then see the beautiful workings of the "similia similibus," to escape the infernal pit by antidotal brimstone! To consume out sin by an infinitesimal application of its ultimate and infinite consequence. To commute the wrath to come into a pellet to swallow! Sin is itch, and the devil is "Old Scratch." Regeneration is the effect of physic—virtue is but health of body. Spiritual christianity is a fiction; its sublime and glorious truths, its soul inspiring revelations, are, correctly understood, only medical expositions of a special and hitherto unmentionable malady. The *Bible is a treatise on cutaneous eruptions!* Similia similibus! is the Shibboleth of salvation—original sin may be counteracted by actual transgression, or by infinitesimal sulphur!

We have long been familiar with the dreadful dilapidation of the human soul. The history of our race furnishes but too many melancholy instances of the stupidity and folly of the moral nature of man; its insensibility to the glorious spirituality in the midst of which it dwells, its blindness to the beautiful, its deafness to the true, its incorrigibility to wisdom, its greediness for pravity, its wonderful affinity for lies; but Mr. Everest and his homœopathic friends have uncovered a deeper depth in moral degradation than we had before had knowledge of. That men, educated in our language, and familiar with its literature: professing christianity and even to be teachers of it: that such men should form and promulge such a scheme of moral life and action as this theory of sin by itch and salvation by sulphur: that there could be men willing to commute the glorious religion of Jesus into such a disgusting conceit as this—was certainly beyond all our previous knowledge or imagination of human capability.

We would recommend to Mr. Everest, as a text, the 3d and 4th verses of the 4th chapter of 2nd Timothy, which in our allopathic version, reads thus: "*For the time will come when they will not endure sound doctrine : but after their own lusts shall they keep to themselves teachers having itching ears: and they shall turn away their ears from the truth and shall be turned unto fables.*"

Though we can charitably believe that Rev. Mr. Everest may be fool enough to place some degree of faith in his own theory: we are far from believing such to be the case with many of the homœopathic practitioners who have had the advantage of medical education, and have in former times given evidence of the possession of at least ordinary intelligence. We may pity the teachers with itching ears, but we must denounce and contemn the doctors with itching palms. The man who essays to extract itch from the soul, certainly wants sense, but not necessarily benevolence and honesty; but well informed men who study to extract unrequited coin from the pockets of the ignorant by such pseudo-medical thimble-rig as homœopathy, cannot shelter their knavery behind the plea of imbecility. They have their reward. They get fees. They ride in carriages. They buy houses. Out of a shadow they derive substance. They who crept, can strut. Who tapped humbly at the door of the cottage, may thunder imperiously at the portal of the palace. Who fretted in solitary places over the neglect of merit, may be astonished at the gainful notoriety of bold and impudent confession of inscience. No wonder that the steps of the honest well nigh slip, when they see the prosperity of the quack, that they are envious of the foolish, who are not in trouble as other men, nor plagued like other men: whose eyes stand out with fatness: who have more than heart could wish! But let them beware that they do not yield to the temptation to go and do likewise. Under all this gratified covetousness and vanity, amidst all the gratulation over obscurity escaped, and duns silenced, and tables supplied, and property gathered, there must be a conciousness of degradation outlasting even the conscience of sin: vanity stung by the contempt of the wise rather than gratified by the homage of the foolish: a sense of loss of caste not compensated by the finding of money. Even after the homœopath of this class has succeeded in the imperative task of justifying his conduct to himself: after he has managed so far to turn his brain upside down as to empty it of the elementary knowledge of his profession, and of the expensive moral precepts which sometimes entangle the opinions of the doctor, long after they have died out of the heart of the man: after he has stultified himself by daily stultification of others, until a comfortable equilibrium of folly is established between him and his victims; after all, the vanity which has grown exuberantly in all this ruin, preserves in the man a sensibility which twinges terribly under contempt, and renders the soul susceptible to pain long after it has lost the capacity of healthy reaction and reform.

One of the most curious parts of Dr. Simpson's book, is that which undertakes to give some mathematical notion of the real quantitative value of homœopathic medicines. The calculations seem to have been made with care, and have been submitted to the examination of professional mathematicians who pronounced them correct. This is a subject which the homœopaths carefully keep out of the view of the public, yet the whole gist of the matter is just here. The homœopath has absolutely no reason for his system, except the experience of the operation of what he facetiously calls his *medicines*. It is, therefore, all important to know what these medicines are. It matters not how loudly he may assert the potency of his means, if they are found to be absolutely and necessarily and beyond all possibility of doubt, inert. If a man or a hundred men should combine to administer powerful doses of moonshine and water, and if they should record cases of recovery under this treatment, as doubtless they might do, it would be impossible to convince reasonable men that the moonshine was the curative potency. It would be in vain for the moonopath to urge that we could know nothing of the medical virtue of swallowed moonshine except by experience. Common sense would say you must first of all prove that you really administered moonshine; and then that the consequences claimed for it, might not have resulted without it.

The homœopath must, in like manner prove, first that he gives medicine at all, and next, that the given medicine produces the effects consequent.

Now we do not hesitate to say, that the homœopath who gives homœopathic preparations, actually gives nothing more than the vehicle assumed to convey the potency. There is a philosophical sense in which this assertion may appear as a mere matter of verbal criticism to be, perhaps, doubtful. We believe it is strictly true in a proportion of experiments so vastly great as to amount almost to universal fact.

Homœopathic dilutions are prepared upon the principle, that matter is not only infinitely divisible, of which there is no proof, and can be none, but that substances soluble in water are infinitely so, and that those capable of mixture with sugar, can never be so intimately mixed as to be incapable of more intimate mixture, by ordinary processes. That is, that the particles of one and the other can never be reduced to such tenuity as not to be made to penetrate and separate each other under the ordinary mechanical attrition of the pestle and mortar. To make the matter more clear, the homœopathic principle being true, a grain of sugar thrown into the ocean, would be so divided that every drop of water in the ocean would hold some of it in solution, and that this would be the case were the ocean a million of million times greater than it is. Again, to believe in the presence of an infinitesimal quantity of physic in a homœopathic globule, one must admit that a grain of sulphur could be rubbed up with a globe of sugar, vastly greater than this earth and that every grain of the mass would be impregnated with brimstone.

We must believe as the first step towards homœopathic faith: afterwards will come the more monstrous proposition that a grain of any substance thus divided endows every grain of the immeasurable mass with medicinal potency! No man ever did or ever can believe this absurdity. Insanity itself cannot degrade the understanding to accept it. Those who honestly profess to believe, only cannot comprehend the meaning of what they profess to believe. It is impossible to force reason beyond the limits of physical possibility; once there, she must shrink from investigation or owning her helplessness, lean upon God and assume the name and character of *faith.* Such faith in homœopathy can only be warranted by Divine revelation. Destitute of this, it presents itself a baseless, senseless, impudent presumption, which can only be tolerated in proportion that it is not understood.

To the homœopath who asserts that there is any fragment of medicine in his infinitesimal preparations, we say *prove it.* Prove that your triturating and shaking has diffused your solitary drop or grain through those immeasurable proportions of sugar, or alcohol, or water. Prove that it has not been precipitated, exhaled, chemically combined; or that it has not reached a point in tenuity which cannot be effected by your mechanical force; not presenting volume enough to be compressed between pestle and mortar; not offering resistance enough to be penetrated by a particle of sugar infinitely greater than itself.

The chances are infinitely great that some such bar to unlimited diffusion has occurred, even if there be no point in the division of matter when the repulsive power of an atom is greater than aggressive force, or where the attraction of cohesion can no longer be overcome by any mechanical power, much less by such gross applications as a pestle millions of millions of times greater than the particle to be rubbed by it, against the sides of a mortar, the most polished sides of which would offer openings, which, compared to a decilionth exiguity, would be mammoth caves. We are warranted, therefore, in at least assuming the negative, that any particular homœopathic pill contains the infinitesimal medicament asserted, and they who assert the fact must *prove it.* We deny that they administer medicine. Let them show the contrary.

Hahneman asserts, that if a desponding and suicidal patient will smell merely a *quintillionth* of a grain of gold, he will be restored within an hour to peace of mind and love of life. Dr. Simpson shows that a grain of gold would be reduced to a quintillionth only by mixing it with a mass equal to sixty-one globes the size of this earth. But Hahneman used much "higher potencies" than quintillionths or fifteenth dilutions. The decilionth or thirtieth dilution was the favorite one. To reduce, says Dr. Simpson, a grain of gold to this decilionth or thirtieth dilution, would require the single grain to be mixed perfectly with a globe of sugar represented by sixty-one quintillions of spheres of sugar, each of these 61,000,000,000,000,000,000,000,000,000,000, being as large as this earth!

Surely it is not worth while to pursue these calculations further! Absurdity, utter, unspeakable absurdity like this, has no comparative degree. The man who can believe that a pill is medicated which he supposes to contain a proportion of medicine to its size, as small as a grain, if it would bear to 61,000,000,000,000,000,000,000,000,000,000 earths, could believe it as well if the proportion were indicated by 60,000 ciphers.

Following out the arithmetical exposition, Dr. Simpson shows that the pill, moistened with the thirtieth dilution, which Hahneman asserts to be indubitably the best dose, consists of a minute globule of sugar, simply dipped in a drop out of an ocean of fluid one hundred and forty billions of times as large as the whole planetary system, and which enormous ocean has been medicated for the purposes of homœopathy by having dissolved and mixed through it one single grain of the appropriate drug.

Such nonsense out-bedlams bedlam. Dr. Henderson does not deny the accuracy of these calculations, but says he knew nothing of the powers of *medicine but by experience.* But what has medicine to do with this matter? Experience of medicine supposes experiment with medicine; experiment with medicine requires the existence and presence, and applicability of medicine. Where is the medicine? In the dried surface of a little pill, once dipped in a solution in which the supposed medicament exists in the proportion of a drop of water in a hundred millions of oceans, each large enough to drown the universe? Do you tell us, that in spite of all reason and common sense, there is medicine in that? Then prove it.

Of Dr. Henderson's reply to Dr. Simpson we have little more to say than that it is no reply at all. It is a poor attempt to divert the attention of the public from the real questions at issue. A large part of it is taken up with the biography and laudation of Hahneman; a considerable part more in sneers and slurs at medicine under the nickname of allopathy and as little as possible upon homœopathy itself. No one from reading the title could guess the character of the book. It is a perfect "lucus a non lucendo;" a title which informs us what the book is not. It is worthy of about as much consideration as is commonly given to the response of the convicted criminal to the rather impertinent inquiry of the judge as to his objections to being hanged. Of course he has objections, but they are not to be supposed to be reasons, nor are they expected to work change in the verdict of the jury or the purpose of the judge. They may be some relief to the culprit, and some comfort to his friends. The last words of Dr. Henderson, have given vent to some bitterness, and may in some way convey, though we do not perceive how, a cordial to his friends. The quantum, if so, is certainly homœopathic.

